# Fatal *Balamuthia mandrillaris* infection with red plaques on the nasal dorsum as the first presentation^⋆^

**DOI:** 10.1016/j.abd.2021.12.001

**Published:** 2022-06-09

**Authors:** Kang Tao, Ting Wang, Lian Zhang, Xi-Chuan Yang, Zhi-Fang Zhai

**Affiliations:** Department of Dermatology and Venereology, Southwest Hospital, Army Medical University, Chongqing, China

**Keywords:** *Balamuthia mandrillaris*, Amebiasis, Encephalitis, Granuloma, Skin diseases

## Abstract

*Balamuthia mandrillaris* infection is a rare infectious disease around the world, with high rates of morbidity and mortality. Its early and correct diagnosis is a big challenge for us, and without it the delay in starting effective treatment can lead to the development of encephalitis. This is a report of a case of *Balamuthia mandrillaris* infection in a Chinese boy, with red plaques on the nasal dorsum as the first presentation, who finally developed into fatal encephalitis. The authors have reviewed the related literature and share the special skin features in order to favor the early diagnosis of the disease and increase the chances of survival.

## Introduction

*Balamuthia mandrillaris* infection is a rare and fatal disease caused by *B. mandrillaris* amoeba, which was first isolated from a pregnant mandrill monkey died from encephalitis in 1986.^1^ It causes Central Nervous System (CNS) disease called Granulomatous Amebic Encephalitis (GAE). Most patients rapidly developed fatal encephalitis, and the lethality rate was up to 90% in the United States from 1974 to 2016.^2^ The epidemiology of *B. mandrillaris* has been little known, and it can only be concluded that soil and polluted water exposures were mentioned frequently in the cases.^3^

## Case report

**Figure 1:**
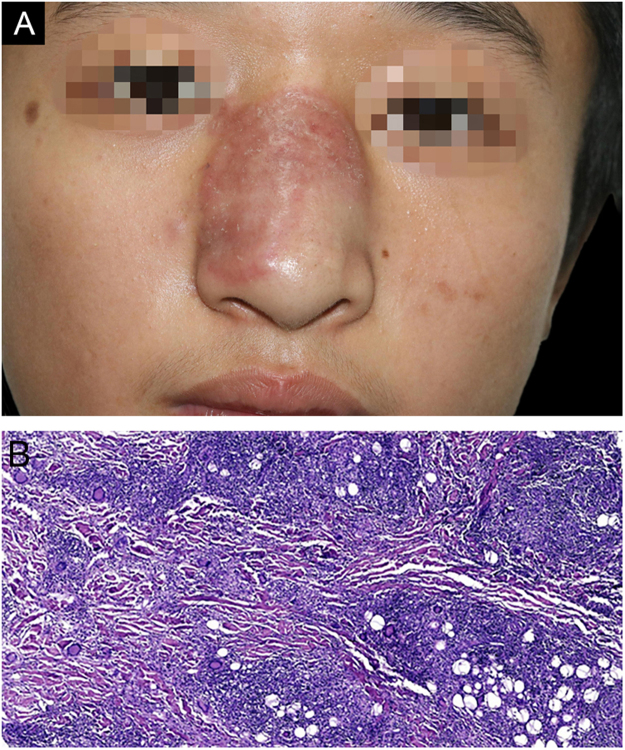
(A), Clinical image showing a red well-circumscribed plaque on his nasal dorsum with slightly raised border and a few scales on the surface. (B), The histopathology examination of the first biopsy shows cutaneous mixed inflammatory cells infiltration, including lymphocytes, histocytes, eosinophils and multinuclear giant cells, with numerous granulomas observed. (Hematoxylin & eosin, ×100).

Here, the authors reported a 15-year-old boy presented with a red plaque on the nasal dorsum over one month, with slight itching. He denied fever or taking any medicine lately. His family and travel history were normal, but he used to swim in the wild pond with ambiguous trauma on the nose. Dermatologic examination revealed a well-circumscribed red plaque with a slightly raised border and mild infiltration with a few scales on the surface (Fig. 1A), involving about two-thirds of his nasal dorsum. No enlarged cervical lymph node was detected.

**Figure 2:**
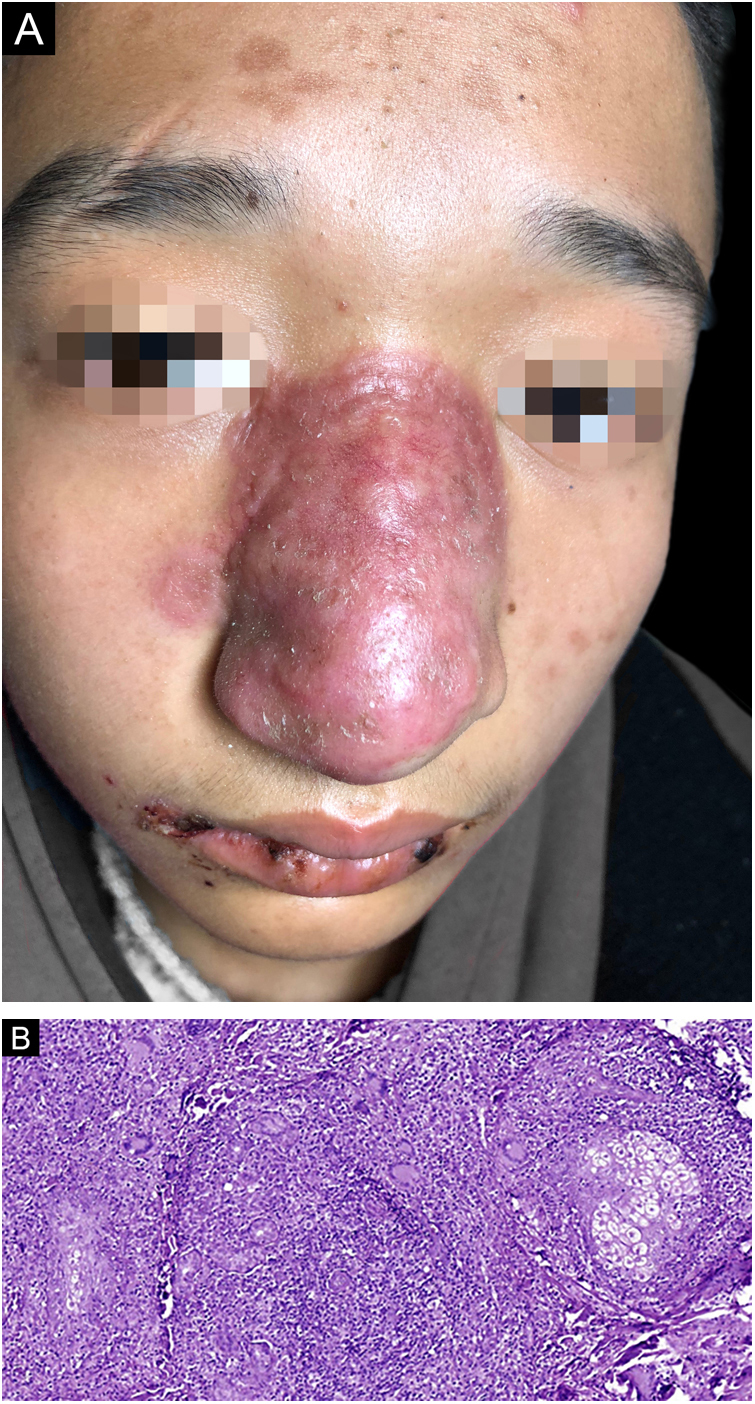
(A), The red plaques enlarged and involved almost the whole nasal dorsum when the patient visited again; (B), The histopathology of the second biopsy showed a similar histological profile as the first biopsy (Hematoxylin & eosin, ×200).

The biopsy was taken from the red plaque, and the histopathology showed mixed inflammatory cells infiltration with the predominance of lymphocytes, histocytes, eosinophils, and multinuclear giant cells, with numerous granulomas observed (Fig. 1B). However, both Periodic Acid-Schiff (PAS) and acid-fast staining were negative, as well as fungal culture and atypical mycobacteriosis detection. Even so, oral Itraconazole was prescribed, 200 mg/day. A month later, he had a “cold” with a slight cough and an enlarged lesion involving almost the whole nasal dorsum (Fig. 2A). Computed Tomography (CT) scan of the lungs was negative, and a second biopsy showed a similar histological profile (Fig. 2B). As the investigation of *Mycobacterium tuberculosis* on lesion sample by PCR-reversed dot hybridization had been negative, the treatment was switched to oral clarithromycin 500 mg/day and doxycycline 200 mg/day, combined with Itraconazole 400 mg/day. Another month later, high-grade fever and repeated nosebleeds occurred. He got into a coma and was hospitalized in another hospital five days later. A cranial CT scan showed multiple abnormal low-density foci in bilateral frontal, parietal lobes, and right occipital lobes. His cerebrospinal fluid sample was sent to Guangzhou Vision medicals Inc. for pathogen detection via Next-Generation Sequence (NGS), which confirmed the presence of *B. mandrillaris* genomic sequences expressed as 88.17% of the whole parasites, which suggests that *B. mandrillaris* was the main pathogen. Though Itraconazole and Linezolid intravenously were given, he died about 2-weeks later, unfortunately. The authors confirmed that *B. mandrillaris* infection already existed in the embedding skin biopsy tissue from initial red plaques by NGS.

## Discussion

Only about 7 cases of *B. mandrillaris* infection were reported per year worldwide.^4^ The patients can be divided into two types by different clinical manifestations. One type is mainly from the United States, which presents with encephalitis without skin lesions, and usually dies within months.^2^, ^4^ The other one is observed mainly in China^5^ and Peru,^6^ where the skin is usually involved first and then gradually develops into encephalitis. Up to 50% of cases were children.^7^ Wang et al. reported 28 Chinese cases, most of them seemed to be less aggressive with higher survival rate (44%), which may be related to the timely treatment before infection developed into GAE. Similarly, the present case also presented with skin lesions, and it took about half a year before encephalitis. Unfortunately, the authors didn't diagnose in time and prevent the infection from rapidly worsening.

It is difficult to diagnose *B. mandrillaris* infection early. Traumatic history and contact history of dirty water or soil should be of concern. Most cases showed a common indurate plaque without ulceration or pustule secretion.^5^ Microscopically, the amoeba looks like histiocytes in morphology and is difficult to be identified. Conventional PAS and acid-fast staining are unhelpful, and the infection is easily misdiagnosed as lethal midline granuloma.^8^ Immunohistochemical staining with anti*-Balamuthia* antibody is useful, while the antibody is not commercially available yet. The next-generation sequence technique is recommended, which can get a higher positive rate for more kinds of microbes, especially some rare pathogen, from different samples, such as tissues, blood, body fluids, and secretions.

The treatment is troublesome for physicians, and there is no standard strategy till now. The miltefosine may benefit patients and should be considered in any treatment plan.^9^ Besides, lincomycin, azithromycin, and interferon-γ may be useful.^5^ Except for drug treatments, Doyle et al. reported a case successfully treated with excision of the infected brain tissue,^10^ but it is not the first choice for most encephalitis. Clinically it is imperative for us to make early diagnoses and discover effective treatments to save more lives.

## Financial support

Our project was supported by the National Natural Science Foundation of China (Grant No.81773316).

## Authors' contributions

Kang Tao: Conception and planning of the study; critical review of the literature; obtaining, analysis, and interpretation of the data; elaboration and writing of the manuscript; critical review of the manuscript; approval of the final version of the manuscript.

Ting Wang: Approval of the final version of the manuscript; obtaining, analysis, and interpretation of the data; critical review of the manuscript.

Lian Zhang: Approval of the final version of the manuscript; obtaining, analysis, and interpretation of the data; critical review of the manuscript.

Xi-Chuan Yang: Approval of the final version of the manuscript; obtaining, analysis, and interpretation of the data; critical review of the manuscript.

Zhi-Fang Zhai: Conception and planning of the study; critical review of the literature; obtaining, analysis, and interpretation of the data; elaboration and writing of the manuscript; critical review of the manuscript; approval of the final version of the manuscript.

## Conflicts of interest

None declared.
